# Unique Internet Search Strategies of Individuals With Self-Stated Autism: Quantitative Analysis of Search Engine Users’ Investigative Behaviors

**DOI:** 10.2196/23829

**Published:** 2021-07-06

**Authors:** Eldad Yechiam, Elad Yom-Tov

**Affiliations:** 1 Faculty of Industrial Engineering and Management Technion - Israel Institute of Technology Haifa Israel; 2 Microsoft Research Herzliya Israel

**Keywords:** autism, decision making, exploration, search, internet

## Abstract

**Background:**

Although autism is often characterized in literature by the presence of repetitive behavior, in structured decision tasks, individuals with autism spectrum disorder (ASD) have been found to examine more options in a given time period than controls.

**Objective:**

We aimed to examine whether this investigative tendency emerges in information searches conducted via the internet.

**Methods:**

In total, 1746 search engine users stated that they had ASD in 2019. This group’s naturally occurring responses following 1491 unique general queries and 78 image queries were compared to those of all other users of the search engine. The main dependent measure was scrolled distance, which denoted the extent to which additional results were scanned beyond the initial results presented on-screen. Additionally, we examined the number of clicks on search results as an indicator of the degree of search outcome exploitation and assessed whether there was a trade-off between increased search range and the time invested in viewing initial search results.

**Results:**

After issuing general queries, individuals with self-stated ASD scanned more results than controls. The scrolled distance in the results page of general queries was 45% larger for the group of individuals with ASD (*P*<.001; *d*=0.45). The group of individuals with ASD also made the first scroll faster than the controls (*P*<.001; *d*=0.51). The differences in scrolled distance were larger for popular queries. No group differences in scrolled distance emerged for image queries, suggesting that visual load impeded the investigative behavior of individuals with ASD. No differences emerged in the number of clicks on search results.

**Conclusions:**

Individuals who self-stated that they had ASD scrutinized more general search results and fewer image search results than the controls. Thus, our results at least partially support the notion that individuals with ASD exhibit investigative behaviors and suggest that textual searches are an important context for expressing such tendencies.

## Introduction

Characterizing the internet browsing style of specific populations is important for understanding how individuals behave during naturally occurring circumstances and can ultimately be used for tailoring interfaces and content to people’s diverse styles and capabilities [1,2]. This paper focuses on the internet search strategies of individuals with autism spectrum disorder (ASD)—a heterogeneous condition characterized by restricted interests and behaviors and by deficits in social communication.

Laboratory studies have discovered that high-functioning individuals with ASD tend to make selections from assorted options in a relatively short period of time [3-7]. For example, Johnson et al [3] examined the performance of adolescents and young adults with ASD in the Iowa gambling task [8]—a 4-choice, repeated, decision-making task. Individuals with ASD and typically developing controls had similar rates of selection with respect to the four options, but those with ASD switched options more frequently than controls. Similarly, in a simulated foraging task, Pellicano et al [5] found that children with ASD used more varied search paths across trials than those of control children. Herein, we analyze data from a large-scale data set and examine whether the heightened propensity for investigation in individuals with ASD emerges when they search for information on the web.

The internet has been described as a “newly autism-compatible environment” [9] due to fact that it is highly suitable for pursuing restricted interests [10] and because it allows individuals to control the mode of social communication [11]. Potentially, when searching for information in this environment, individuals with ASD may express their investigative tendencies by thoroughly examining search results. Alternatively, people with ASD tend to be more distracted by novel stimuli than typically developing persons [12,13]. They also tend to avoid situations involving multiple decisions [14,15]. If the novel stimulus and choice overload associated with ASD are sufficiently strong, they may restrict the examination of search results.

To discern whether individuals with self-stated ASD (s-sASD) examine internet search results more thoroughly than individuals without ASD, we analyzed their naturally occurring interactions with an internet search engine. Our research strategy was different from that of most autism studies, which typically use small samples of psychiatrically diagnosed participants [10,16,17]. We obtained historical browsing records for a very large group of users who stated that they had ASD in one of their search queries. We then used the same data set to filter identical queries made by users who did not refer to themselves as having ASD. Therefore, for a given query, we could compare the two groups’ responses to the same query results and thus control for the content of the query (as well as any implied personality and cognitive style differences) and the specific search results. For robustness, we examined browsing records for two major domains—general web searches (study 1) and image searches (study 2).

Our main dependent variable was the scanned range of search results, that is, the extent to which individuals chose to present themselves with more results in addition to those shown on the initial results screen. In addition, we examined the number of clicks on search results (links to other pages) as an estimate of the exploitation of search outcomes. Finally, we tested whether there was a trade-off between increased search range and the time invested in viewing initial search results.

## Methods

### Study Design

This study was preapproved by the Technion Research Ethics Committee (approval number: 2020003). The data set used in this study is proprietary to Microsoft Corporation and was kept anonymous for privacy reasons. This data set was approved for research, which was to be conducted by the second author in his capacity as a Microsoft Research Senior Principal Researcher. By using a data set of all English-language queries made by people in the United States on the Bing search engine in 2019, we extracted each user’s anonymized ID and their query text. Queries were filtered to identify users who described themselves as autistic in one of their queries. This was determined by the usage of one of the following phrases in query text: *I have autism*, *I’m autistic*, *I’m on the autism*, *I am on the autism*, and *my autism*. Queries that suggested that a user was unsure (eg, *do I have autism*) were excluded from the analysis. In addition, we excluded queries in which there was information suggesting that the reference to autism did not characterize the user (eg, *I have autism hat*). A complete list of exclusion terms appears in Multimedia Appendix 1. We referred to the group of users that we identified in this manner as individuals with s-sASD in 2019. The number of users in this group was 1746. The control group included all other users who were not identified as individuals with s-sASD and made queries on Bing in the United States in 2019.

### Study 1: General Web Search

In study 1, we targeted all general web searches in Bing made by individuals with s-sASD in November 2019. Additionally, we examined identical searches that were made by the control group during the same time period. Our main dependent variable was the total distance scrolled (in pixels) in the search results screen, which denoted the extent to which additional results were scanned after the initially presented, on-screen results were scanned. For validation purposes, we also compared the number of scroll events. Scroll events are discrete increases in search range that have unspecified lengths and are produced by the user (using a keyboard or mouse). Additionally, we studied the number of clicked links in the search results. Furthermore, we compared the two groups’ response times, that is, the amount of time until the first scroll event (ie, before the search range was increased) and, for control purposes, the time to the first mouse movement. We included unique queries that were searched at least 10 times. Additionally, we only included queries that were followed by mouse movements, which denoted a user’s response. The total number of queries was 1491, and this represented over 300 million searches; 37,810 were conducted by individuals with s-sASD, and the rest were conducted by the control group.

### Study 2: Image Search

In this study, we targeted all image searches in Bing made by individuals with s-sASD in November 2019 and all matching image searches made by the control group. Initially, in study 2a, we used the same search breadth variables as those in study 1—the total distance scrolled (in pixels), number of scroll events, and number of image clicks. As the criterion of conducting 10 or more searches for a unique query produced a small amount of queries, we included unique image queries that were searched at least 5 times by both the study and control populations. Additionally, as previously stated, we only included queries that were followed by mouse movements. The total number of matching queries was only 38, which represented 364,386 searches (258 made by individuals with s-sASD). The relatively small number of matching image queries was likely due to the exclusion of devices such as tablets and cellphones, which do not have a mouse. In order to broaden the sample, in study 2b, we used an alternative dependent variable—the number of thumbnail images displayed to the user. This is similar to the total distance scrolled; however, it also includes the initial number of images that are available before scrolling. In this substudy, we also included searches with no mouse movements. The total number of image queries in study 2b was 78 (approximately 1.6 million searches, of which 698 were conducted by individuals with s-sASD).

### Analysis

Search indices were averaged across each unique query. Each unique query therefore had a pair of data points—one data point set from the group of individuals with s-sASD and one from the control group. Query topic categories were identified by a proprietary classifier. Age and gender data were also available for a subset of users who were registered with Bing. Our main analysis involved a comparison of the differences between groups in terms of search indices across queries (using two-tailed paired *t* tests). This analysis was independent of the number of times that each query was issued. The analysis was therefore not confounded by the query topic because it examined responses that were contingent on a given query. In addition, to account for the effect of the popularity of searches, we also compared the average number of individual searches by multiplying the search parameters of each query by its relative volume (ie, the number of times that the query was issued divided by the average number of times a query was issued in a given group). If the difference between groups per query was not the same as the difference between individual searches, this implied that the volume of searches affected the gap between the two groups. In other words, this implied that the difference between groups was moderated by query popularity. The two differences were compared by using a repeated measures analysis of variance, in which the groups and units of analysis (queries vs searches) were paired factors.

## Results

### Search Queries

To further validate our self-statement method, we examined all Bing searches conducted by individuals with s-sASD in 2019 that included the words *autism* or *autistic*. On average, there were 14.17 such searches (SE 1.14). For comparison, we also examined those in the control group who made at least 1 search with the words *autism* or *autistic*. Their average number of relevant searches was only 2.52 (SE 0.003), and the difference in the average number of relevant searches between the two groups was highly significant (rank sum *P*<.001).

The top query topics of the individuals with s-sASD and the control group in November 2019 are shown in Table 1. Individuals with s-sASD were more likely to query about books and consumer electronics than controls and were more interested in images of automobiles and video games. In contrast, the control population made more queries related to travel and flights. We further examined whether the popularity of different queries was similar between the two groups (individuals with s-sASD and controls). The correlation between the volume of general queries included in this study for the two groups was 0.53 (*P*<.001), and for image searches, the correlation was 0.02 (*P*=.86). This implied that the relative search popularity of general queries was highly similar between groups, but this was not true for image-related queries.

**Table 1:** The top 3 query topics that were distinctly popular in either the group of individuals with s-sASD or the control group during November 2019. Odds ratios (ORs) and 95% CIs are presented.

Topics				OR (95% CI)
**Topics that were more popular among individuals with** **self-stated autism spectrum disorder**				
	**General queries**			
		Television shows	8.83 (8.49-9.18)	
		Books	6.16 (5.73-6.63)	
		Consumer electronics	4.36 (3.95-4.80)	
	**Image queries**			
		Video games	4.20 (3.09-5.72)	
		Things to do	2.73 (2.42-3.08)	
		Automobiles	2.70 (2.46-2.95)	
**Topics that were more popular in the control group**				
	**General queries**			
		Flights	4.82 (4.11-5.66)	
		Travel guide	4.70 (4.19-5.27)	
		Things to do	4.47 (4.04-4.94)	
	**Image queries**			
		Travel guide	7.08 (5.52-9.10)	
		Restaurants	2.96 (2.50-3.52)	
		Television shows	1.69 (1.53-1.86)	

### Study 1: General Search

**Figure 1:**
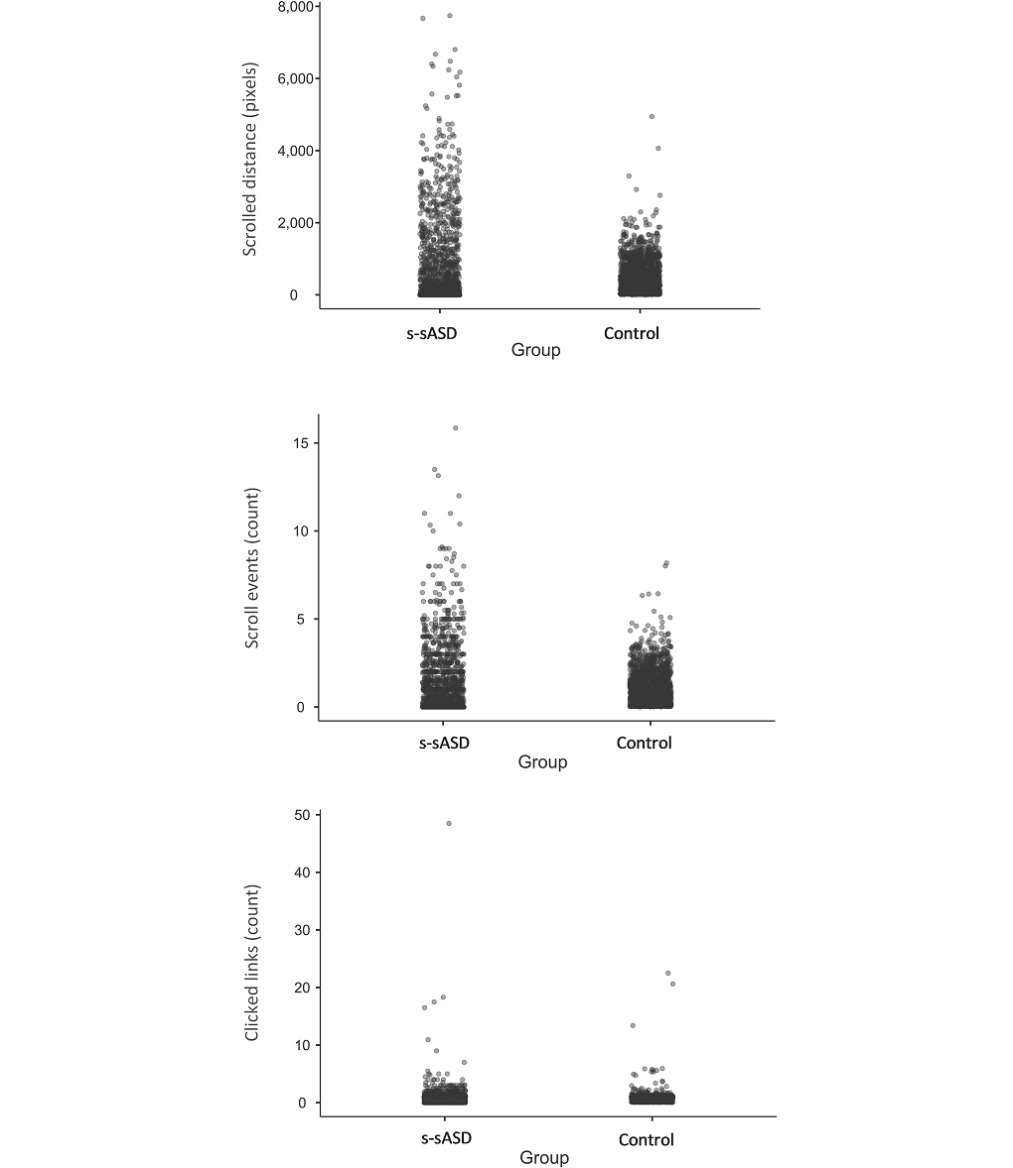
Data for study 1 (search breadth indices stratified by group). The mean scrolled distance, number of scroll events, and number of clicked links for each query are presented. s-sASD: self-stated autism spectrum disorder.

Table 2 shows the mean values of search parameters for the two groups, and Figure 1 presents the distribution of search parameters. Our main analysis of average values across different queries indicated that the scrolled distance was about 1.45 times higher among individuals with s-sASD (*t*_1490_=8.63; *P*<.001; *d*=0.45), and the number of scroll events was about 1.12 times higher (*t*_1490_=3.29; *P*=.001; *d*=0.17). In contrast, differences in the number of clicked links were smaller and not statistically significant (*t*_1490_=1.91; *P*=.06; *d*=0.10).

**Table 2:** Study 1 results: the mean values of general search parameters for individuals with self-stated autism spectrum disorder (s-sASD) and the control groups.

Variables			Values per query^a^, mean (SE)				Average per individual search^b^, mean (SE)				
			Individuals with s-sASD		Controls (matched)		Individuals with s-sASD		Controls (matched)		Controls (all other users)
**Search breadth variables**											
	Scrolled distance (pixels)	745.38 (30.62)		513.44 (12.66)		835.29 (54.81)		136.07 (12.10)		231.16 (9.05)	
	Number of scroll events	1.33 (0.05)		1.18 (0.03)		1.35 (0.08)		0.32 (0.02)		0.55 (0.02)	
	Number of clicked links	0.94 (0.04)		0.86 (0.03)		1.11 (0.10)		0.93 (0.20)		0.86 (0.10)	
**Search time variables (s)**											
	Time to first scroll	35.99 (0.59)		40.81 (0.30)		33.83 (2.88)		51.34 (9.48)		48.74 (4.96)	
	Time to first mouse movement	3.28 (0.12)		3.91 (0.05)		3.21 (0.23)		4.48 (0.97)		4.14 (0.36)	

^a^The average values for each query give the same weight to each query.

^b^The average values for each individual search were weighted by the relative volume of searches for each unique query.

**Figure 2:**
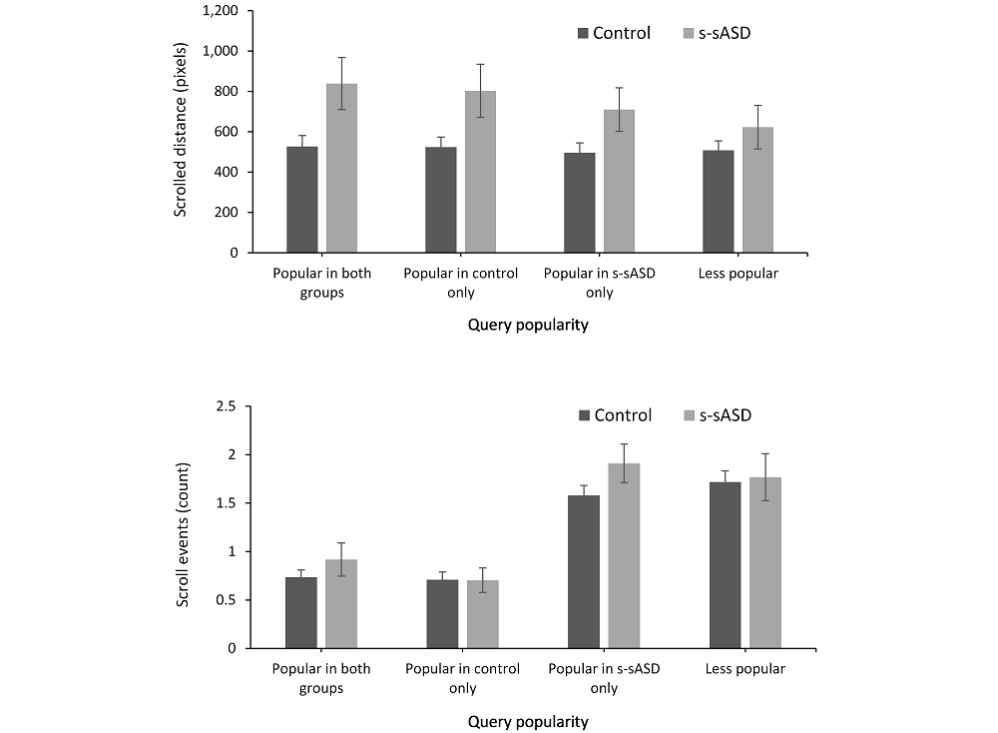
The effect of relative search volume on differences between groups in study 1. Queries were divided into the top 50% of queries in both groups, those in the control group, those in the group of individuals with s-sASD, and those in neither group. The error bars denote 95% CIs. s-sASD: self-stated autism spectrum disorder.

The search breadth indices of the two groups were even more distinct when we compared individual searches (Table 2). The difference between the two groups’ individual searches was significantly larger than the differences of each query in terms of scrolled distance (*F*_1,1490_=98.36; *P*<.001) and the number of scroll events (*F*_1,1490_=154.71; *P*<.001). However, this was not true for the number of clicked links (*F*_1,1490_=0.36; *P*=.54). The scrolled distance of individual searches conducted by individuals with s-sASD was 6.14 times higher than that of the control group’s searches (*t*_1490_=13.91; *P*<.001; *d*=0.72), and the number of scroll events was about 4.28 times higher (*t*_1490_=12.51; *P*<.001; *d*=0.71). This indicated that the queries’ popularity moderated the differences in search breadth between groups, and the difference was higher for popular queries. To illustrate this effect, in Figure 2, we depict the differences in search indices among the top 50% of queries of both groups, those only in the control group, those only in the group of individuals with s-sASD, and those in neither group. As can be seen in Figure 2, a large difference between groups in terms of scrolled distance emerged in queries that were popular in both groups, whereas a smaller difference emerged for queries that were relatively unpopular. Similarly, the difference between groups in terms of the number of scroll events mostly emerged for queries that were popular among individuals with s-sASD.

Table 2 also presents the average response times of the two groups. Individuals with s-sASD were considerably faster at making the first scroll event (*t*_1490_=9.93; *P*<.001; *d*=0.51) and moving the mouse after conducting searches (*t*_1490_=4.97; *P*<.001; *d*=0.26), suggesting that there was some trade-off between search breadth and the time invested in scanning the initial search results.

The differences between groups seemed consistent with the notion that ASD is associated with increased investigative behaviors. However, an alternative interpretation is that these differences were due to a demographic disparity between groups. Populations with ASD typically have a gender ratio of about 4:1 (males to females) [18]. Thus, it was important to determine if our results were moderated by gender. Since gender information was available only for a small subsample of users (less than 0.5% of the total users), we focused on participants in the control group and examined the effects that gender (0=female; 1=male) and age had on search indices by using linear regressions. A total of 5179 queries (approximately 7.0 million searches) were available for this analysis. The findings are summarized in Table 3. As can be seen in Table 3, gender had a rather small effect on the breadth of searches. Males’ scrolled distance was higher than that of females by about 19 pixels, which equals 3.6% (pixels: 18.91/513.44) of the average scrolled distance of the control group. In contrast, the effect of s-sASD amounted to 45.2% (pixels: 231.94/513.44 pixels) of the average scrolled distance of the control group (pixels: 231.94/513.44). Thus, it appeared that the difference between genders was considerably smaller than the effect of self-stated autism.

Finally, because we matched the control group’s queries to those of individuals with s-sASD, the studied queries represented 48.7% of the searches conducted by those with s-sASD and 15.2% of the searches conducted by the control group. In order to ensure that the subsample of searches conducted by the control group was not biased, we extracted all unique queries that were searched more than 10 times in the control group, except those that were included in our original sample (a total of 23,071 unique queries). We focused on indices for individual searches, since these queries were different from those included in this study. As can be seen in Table 2, the search breadth indices of all remaining queries in the control group were somewhat higher than those included in this study; however, they were still far below those recorded for the individuals with s-sASD.

**Table 3:** Study 1 results: an examination of the effects of age and gender in the control group. The adjusted r2 denotes the fit of the regression model (proportion of explained variance).

Predicted variables	Age, unstandardized coefficient (SE)	Gender, unstandardized coefficient (SE)	Adjusted *r*^2^
Scrolled distance	0.07 (0.02)	18.91 (0.71)	0.004^a^
Scroll events	0.0008 (0.0001)	0.05 (0.002)	0.02^a^

^a^Significant at the *P*<.001 level.

### Study 2: Image Search

**Figure 3:**
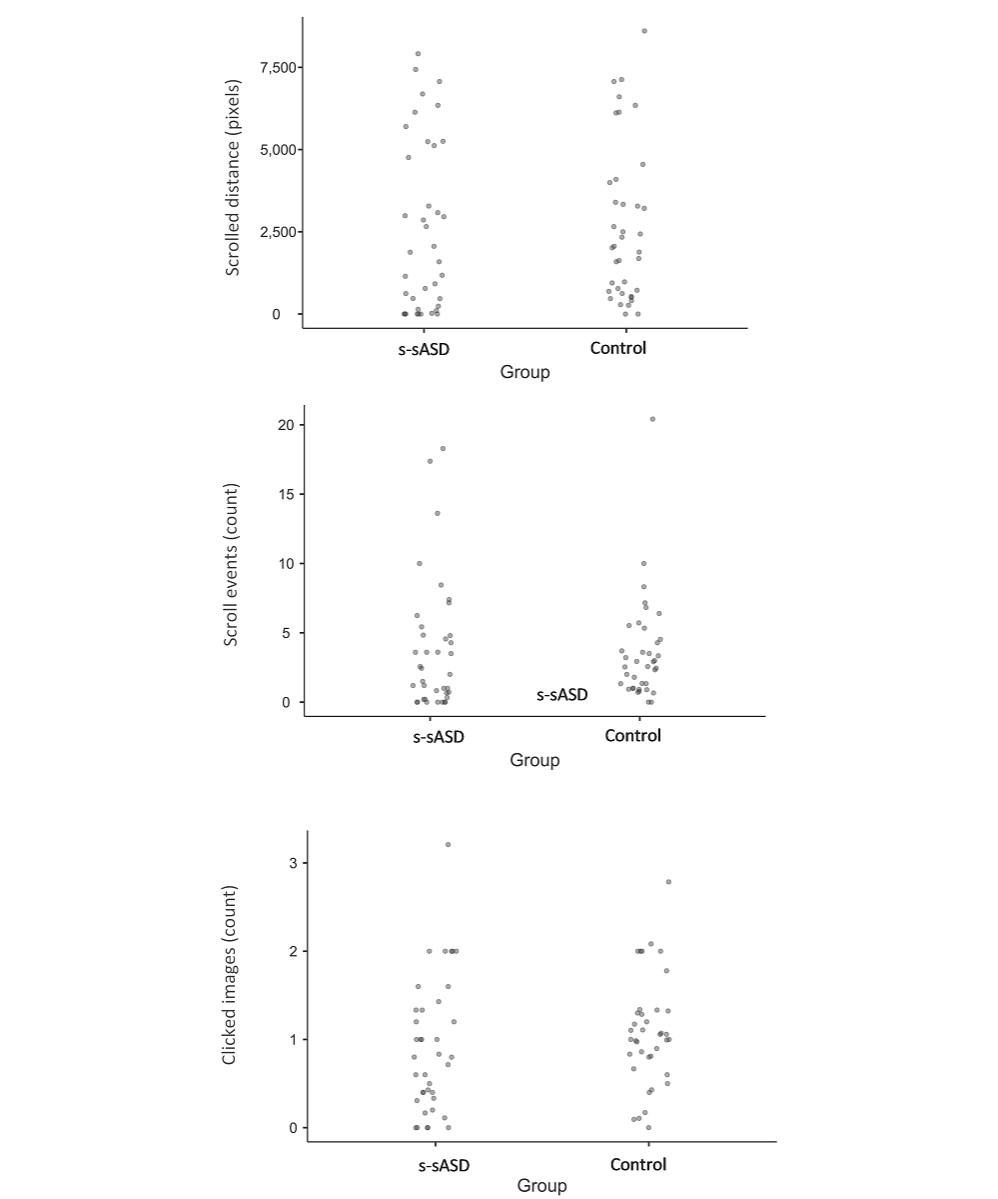
Data for study 2a stratified by group. The mean scrolled distance, number of scroll events, and number of clicked images for each query are presented. s-sASD: self-stated autism spectrum disorder.

Table 4 shows the mean values of image search parameters for the two groups, and the distribution of search parameters is presented in Figure 3. Different from study 1, in study 2a, we found that the scrolled distances were about equal between the two groups (*t*_37_=0.43; *P*=.67; *d*=0.14). This was also true for the number of scroll events (*t*_37_=0.32; *P*=.75; *d*=0.10). Additionally, on average, the group of individuals with s-sASD clicked on images considerably less than the control group (*t*_37_=2.81; *P*=.008; *d*=0.91).

As in study 1, the tendency of participants with s-sASD to exhibit greater search breadth was more distinct when considering all individuals’ searches rather than their average per query. This interaction was significant in terms of scrolled distance (*F*_1,37_=9.05; *P*=.005) and the number of scroll events (*F*_1,37_=5.58; *P*=.02). However, this was not true for the number of images clicked (*F*_1,37_=4.05; *P*=.052). As indicated in Table 4, the mean scrolled distance in individual searches conducted by the individuals with s-sASD was higher than that of the control group (*t*_37_=2.39; *P*=.02; *d*=0.78), as was the mean number of scroll events (*t*_37_=2.36; *P*=.02; *d*=0.77). This was due to the fact that, as in study 1, for highly popular queries, individuals with s-sASD scanned more images than controls, and the number of individual searches for these queries was an order of magnitude higher than the median.

Similar findings emerged in study 2b, which included a somewhat larger number of queries (Table 4 and Multimedia Appendix 1). There was no significant difference in the number of images presented to the two groups per query (*t*_77_=0.12; *P*=.90; *d*=−0.03). Additionally, in this large sample, the average number of image clicks in the group of individuals with s-sASD was smaller than that in the control group (*t*_77_=4.43; *P*<.001; *d*=−1.00). The null effect of the number of images was similar when examining the average values for each query and average values for individual searches (*F*_1,77_=2.81; *P*=.10).

We also examined whether the null effect in study 2b was due to queries mainly producing images of faces. We divided the image queries into those including individual persons’ names (eg, “Bjork smiling”: n=21; other queries: n=57) and compared the number of images presented in each category. The results indicated that for people-related searches, the control group was presented with, on average, 101.8 (SE 8.4) images, whereas 88.5 (SE 8.3) images were presented to the group of individuals with s-sASD. In contrast, for nonpeople-related searches, the control group was presented with 89.2 (SE 4.9) images, whereas 95.4 (SE 10.8) images were presented to the group of individuals with s-sASD. However, this crossover interaction trend was not significant in the repeated measures analysis (*F*_1,76_=1.35; *P*=.25).

**Table 4:** Study 2 results: the mean values of image search parameters for individuals with self-stated autism spectrum disorder (s-sASD) and the controls.

Variables			Values per query^a^, mean (SE)				Values per individual search^b^, mean (SE)		
			Individuals with s-sASD		Controls		Individuals with s-sASD		Controls
**Study 2a variables**									
	Scrolled distance	2555.5 (419.5)		2680.5 (384.1)		3,111.7 (299.62)		668.23 (278.39)	
	Scroll events	3.75 (0.75)		3.56 (0.59)		4.43 (1.07)		1.03 (0.43)	
	Clicked images	0.90 (0.12)		1.08 (0.10)		1.09 (0.30)		0.28 (0.14)	
**Study 2b variables**									
	Images displayed	93.51 (8.16)		92.60 (4.24)		93.88 (13.32)		55.10 (17.39)	
	Clicked imaged	0.42 (0.06)		0.73 (0.07)		0.47 (0.11)		0.12 (0.04)	

^a^The average values for each query give the same weight to each query.

^b^The average values for each individual search were weighted by the relative volume of searches for each unique query.

## Discussion

The first major finding we observed was that there was a large difference between how the individuals with s-sASD investigated search results for general searches and image searches. In general searches, participants with s-sASD scanned more results than controls by scrolling down on the screen of results. For example, for general queries, the scrolled distance of participants with s-sASD was 1.45 times larger than that of the control group (745.38 pixels vs 513.44 pixels). In image searches, no such trend emerged. The increased search range in general searches made by individuals with s-sASD supports the validity of laboratory studies showing that high-functioning individuals with ASD are prone to inspective behaviors [5,19]. However, the fact that this phenomenon was not found in such individuals conducting image searches suggests that there is an important (and unexplored) moderator for the propensity to investigate autism.

The differences between image searches and general searches may have been due to the fact that image searches involve greater visual load than typical general searches. In individuals with ASD, the capacity for selective attention was found to be more impaired by high visual load when compared to that capacity in typically developing persons [20]. Additionally, image search results usually include more visual depictions of social interactions and faces, which individuals with ASD may find difficult to process [21-23]. Our findings showed that differences between groups were not significantly affected by whether the query was about an individual person, but additional individual-level analyses are necessary to further validate this finding.

The second major finding of this study was that the investigative tendency of individuals with s-sASD was more pronounced in individuals’ average search parameters (across all of the queries they made) than in parameters averaged at the query level. This interaction was driven by an increase in the gap in highly popular queries between groups. For example, in study 1, with regard to the 20 most frequently searched queries in each group, the scrolled distance of individuals with s-sASD was 2.16 times higher than the mean scrolled distance of the control group. In contrast, with regard to the 20 least frequently performed queries, the scrolled distance for individuals with s-sASD was only about 1.26 times higher. Similar patterns emerged in study 2. The fact that differences between groups emerged mainly for queries that were highly popular among individuals with s-sASD is consistent with the principal tendency of such individuals to make large efforts for relatively specific areas of interest [24,25].

An important limitation of this study is the fact that individuals with s-sASD were not diagnosed with autism. Indeed, the only information about their identity stemmed from the statements that they made while searching for information on the web. Nevertheless, our validation analysis showed that these individuals were also actively making queries regarding autism-related websites. Furthermore, the favorite queries of individuals with s-sASD (Table 1) suggested that these individuals were more interested in technical topics than in social topics. Additionally, we controlled for the possibility that differences between groups might occur due to factors such as age and gender by examining the effect of these factors in the control group. Another limitation of this study is that it presumably represents a subsample of individuals with ASD who are capable of writing and are computer literate. Little is known about the search styles of low-functioning individuals with autism [26].

In our opinion, our findings make two contributions to the study of autism. First, they shed light on how individuals with self-reported ASD engage in naturally occurring internet searches. The answer appears to differ when the search mainly produces text and when it produces images, and these differences are affected by the queries’ relative popularity. Therefore, an interesting open question is whether the web search style of individuals with autism, which involves the fast scanning of many search results, can be adapted by individuals with autism and those without autism.

Second, our findings provide a new method for assessing autism via the “digital footprints” of one’s search statements. Therefore, we transitioned from the use of single or multiple keywords [27,28] to the use of self-referenced descriptors. Although we have relied on data that are not publicly available to achieve this goal, similar data can be extracted through crowdsourcing [29].

## Appendix

Multimedia Appendix 1 List of exclusion terms.

## Abbreviations

ASD: autism spectrum disorder
s-sASD: self-stated autism spectrum disorder

## References

[1] Yom-Tov E, Brunstein-Klomek A, Mandel O, Hadas A, Fennig S (2018). Inducing behavioral change in seekers of pro-anorexia content using internet advertisements: Randomized controlled trial. JMIR Ment Health.

[2] Yoo DW, Birnbaum ML, Van Meter AR, Ali AF, Arenare E, Abowd GD, De Choudhury M (2020). Designing a clinician-facing tool for using insights from patients' social media activity: Iterative co-design approach. JMIR Ment Health.

[3] Johnson SA, Yechiam E, Murphy RR, Queller S, Stout JC (2006). Motivational processes and autonomic responsivity in Asperger's disorder: evidence from the Iowa Gambling Task. J Int Neuropsychol Soc.

[4] Yechiam E, Arshavsky O, Shamay-Tsoory SG, Yaniv S, Aharon J (2010). Adapted to explore: reinforcement learning in Autistic Spectrum Conditions. Brain Cogn.

[5] Pellicano E, Smith AD, Cristino F, Hood BM, Briscoe J, Gilchrist ID (2011). Children with autism are neither systematic nor optimal foragers. Proc Natl Acad Sci U S A.

[6] Mussey JL, Travers BG, Klinger LG, Klinger MR (2015). Decision-making skills in ASD: performance on the Iowa Gambling Task. Autism Res.

[7] Vella L, Ring HA, Aitken MR, Watson PC, Presland A, Clare IC (2018). Understanding self-reported difficulties in decision-making by people with autism spectrum disorders. Autism.

[8] Bechara A, Damasio AR, Damasio H, Anderson SW (1994). Insensitivity to future consequences following damage to human prefrontal cortex. Cognition.

[9] Murray D, Lesser M (1999). Autism and Computing.

[10] Jordan CJ, Caldwell-Harris CL (2012). Understanding differences in neurotypical and autism spectrum special interests through Internet forums. Intellect Dev Disabil.

[11] Gillespie-Lynch K, Kapp SK, Shane-Simpson C, Smith DS, Hutman T (2014). Intersections between the autism spectrum and the internet: perceived benefits and preferred functions of computer-mediated communication. Intellect Dev Disabil.

[12] Gomot M, Bernard FA, Davis MH, Belmonte MK, Ashwin C, Bullmore ET, Baron-Cohen S (2006). Change detection in children with autism: an auditory event-related fMRI study. Neuroimage.

[13] Sokhadze E, Baruth J, Tasman A, Sears L, Mathai G, El-Baz A, Casanova MF (2009). Event-related potential study of novelty processing abnormalities in autism. Appl Psychophysiol Biofeedback.

[14] Luke L, Clare IC, Ring H, Redley M, Watson P (2012). Decision-making difficulties experienced by adults with autism spectrum conditions. Autism.

[15] Gaeth GJ, Levin IP, Jain G, Burke EV (2016). Toward understanding everyday decision making by adults across the autism spectrum. Judgm Decis Mak.

[16] Daly BP, Nicholls EG, Patrick KE, Brinckman DD, Schultheis MT (2014). Driving behaviors in adults with autism spectrum disorders. J Autism Dev Disord.

[17] Levin IP, Gaeth GJ, Foley-Nicpon M, Yegorova V, Cederberg C, Yan H (2015). Extending decision making competence to special populations: a pilot study of persons on the autism spectrum. Front Psychol.

[18] Loomes R, Hull L, Mandy WPL (2017). What is the male-to-female ratio in autism spectrum disorder? A systematic review and meta-analysis. J Am Acad Child Adolesc Psychiatry.

[19] Zeif D, Yechiam E (2020). Autism is not associated with poor or enhanced performance on the Iowa Gambling Task: A Meta-Analysis. Neurosci Biobehav Rev.

[20] Tyndall I, Ragless L, O'Hora D (2018). Effects of perceptual load and socially meaningful stimuli on crossmodal selective attention in Autism Spectrum Disorder and neurotypical samples. Conscious Cogn.

[21] Klin A, Jones W, Schultz R, Volkmar F, Cohen D (2002). Visual fixation patterns during viewing of naturalistic social situations as predictors of social competence in individuals with autism. Arch Gen Psychiatry.

[22] Norbury CF, Brock J, Cragg L, Einav S, Griffiths H, Nation K (2009). Eye-movement patterns are associated with communicative competence in autistic spectrum disorders. J Child Psychol Psychiatry.

[23] Vabalas A, Freeth M (2016). Brief report: Patterns of eye movements in face to face conversation are associated with autistic traits: Evidence from a student sample. J Autism Dev Disord.

[24] Gillberg IC, Gillberg C (1989). Asperger syndrome--some epidemiological considerations: a research note. J Child Psychol Psychiatry.

[25] Ozonoff S (1995). Reliability and validity of the Wisconsin Card Sorting Test in studies of autism. Neuropsychology.

[26] Lambrechts A, Cook J, Ludvig EA, Alonso E, Anns S, Taylor M, Gaigg SB (2020). Reward devaluation in autistic children and adolescents with complex needs: A feasibility study. Autism Res.

[27] Ayers JW, Althouse BM, Allem J, Rosenquist JN, Ford DE (2013). Seasonality in seeking mental health information on Google. Am J Prev Med.

[28] Brigo F, Igwe SC, Ausserer H, Nardone R, Tezzon F, Bongiovanni LG, Trinka E (2014). Why do people Google epilepsy? An infodemiological study of online behavior for epilepsy-related search terms. Epilepsy Behav.

[29] Sadeh-Sharvit S, Fitzsimmons-Craft EE, Taylor CB, Yom-Tov E (2020). Predicting eating disorders from internet activity. Int J Eat Disord.

