# Supplementary material for: Unique Internet Search Strategies of Individuals With Self-Stated Autism: Quantitative Analysis of Search Engine Users’ Investigative Behaviors
Source: J Med Internet Res. 2021 Jul 6;23(7):e23829. doi: 10.2196/23829 (PMC8292935; doi:10.2196/23829)
Supplement: Multimedia Appendix 1 [file jmir_v23i7e23829_app1.docx]

**Multimedia Appendix 1.** List of exclusion terms.

The following terms were used to exclude queries: *I think I’m autistic*, *I have autism card*, *I have autism book*, *my autism connection*, *can I have autism*, *autism child*, *my autism team*, *could I have autism*, *my autism story*, *how can I tell if*, *know if I have autism*, *see if I have*, *how do I know*, *I think I have*, *do I have autism*, *how to tell*, *true life*, *be patient*, *hat*, *song*, *shirt*, and *skirt*.
